# Supplementary material for: Tuning the potency and selectivity of ImmTAC molecules by affinity modulation
Source: Clin Exp Immunol. 2023 Nov 1;215(2):105–19. doi: 10.1093/cei/uxad120 (PMC10847821; doi:10.1093/cei/uxad120)
Supplement: uxad120_suppl_Supplementary_Tables_S1-S5_Figures_S1-S8 [file uxad120_suppl_supplementary_tables_s1-s5_figures_s1-s8.pdf]

Supplementary material

Supplementary Tables

Supplementary Table 1 – **Affinity of anti-CD3 for CD3εδ.** Biacore single-cycle measurements for anti-CD3 variants binding CD3εδ conducted at 37 °C in PBS supplemented with 0.005% surfactant P20.

U number	k_{on}	k_{off}	CD3 binding K_D (nM)	t 1/2 (min)
**E8**	3.21E+04	0.0197	**615**	**0.59**
**E39**	1.18E+05	0.0714	**607**	**0.16**
**E37**	1.08E+05	0.0308	**286**	**0.38**
**E41**	9.38E+04	0.02472	**264**	**0.47**
**E2**	6.56E+04	0.009428	**144**	**1.23**
**E35**	1.02E+05	0.01396	**137**	**0.83**
**E36**	1.03E+05	0.01286	**125**	**0.90**
**E31**	9.01E+04	0.00811	**90**	**1.42**
**E38**	7.07E+04	0.00575	**81**	**2.01**
**E30**	7.39E+04	0.0028	**37.8**	**4.13**
**E28**	6.88E+04	0.00249	**36.2**	**4.64**
**E0**	4.48E+04	0.00152	**33.9**	**7.60**
**E29**	4.75E+04	0.00176	**30.9**	**6.56**
**E16**	1.37E+05	0.000812	**5.91**	**14.23**
**E20**	1.93E+05	0.00107	**5.54**	**10.80**
**E17**	1.60E+05	0.000373	**2.34**	**30.97**
**E22**	2.21E+05	0.000129	**0.58**	**89.55**
**E24**	1.74E+05	0.0000901	**0.52**	**128.22**
**E42**	2.03E+05	0.0000499	**0.25**	**231.51**

Supplementary Table 2 – Affinity of TCRs used in this study for target and mimetic peptides. Kinetics measured by Biacore at 37°C using single cycle method and five injections of tenfold dilutions. Target peptides presented by HLA are listed in peptide and sequence columns, including mimetic peptides with the amino acids that differ from the target highlighted in red. *MimH off-rate was potentially faster than the upper range that could be accurately measured by the instrument.

TCR	Peptide	Peptide seq	HLA	k_{on}	k_{off}	K_D nM	t 1/2 min	Notes
NY-BR-1	TAX	LLFGYPVYV	A2	-	-	-	-	No binding
	MimI	SLIKNLDPV	A2	-	-	566900	-	Equilibrium fit
	MimH	SLMKILSEV	A2	*2.87E+5**	1.89*	65969	*~0.006**	Equilibrium fit + dissociation used to calculate k_{on}
	MimG	SLSIVLSTV	A2	3.85E+05	0.71963	1870	0.02	
	MimF	SLSAILDTV	A2	3.69E+05	0.416805	1131	0.03	
	MimE	SLSKITTTV	A2	5.44E+05	0.218521	402	0.05	
	MimD	SLSKILATV	A2	7.33E+05	0.123204	168	0.09	
	MimC	SLSKIADTV	A2	4.95E+05	0.043133	87	0.27	
	MimB	SLSKALDTV	A2	3.10E+05	0.003494	12.3	3.3	
	MimA	SLAKILDTV	A2	1.06E+06	0.001714	1.84	6.7	
	**NY-BR-1**	**SLSKILDTV**	A2	3.81E+05	0.000156	0.37	74	
**TCR-X**			A2	4.69E+05	0.000035	0.076	325	
**MAG-IC3**	Titin	ESDPIVAQY	A1	1.37E+05	0.02493	183	0.46	
	**MAGE-A3**	**EVDPIGHLY**	A1	1.92E+05	0.003193	16.7	3.6	

Supplementary Table 3 – Parameters and model inputs. Summary of variables input into the mathematical model, including which are fixed input values, and which are fitted to the data.

Parameter	Symbol	Value
Well area		0.32 (96 wells) or 0.056 (384 well)
'Active' volume		3.2000e-08 (96 well) Or 5.6000e-09 (384 well) (1 micron slice x area of well)
ImmTAC concentration		0.1 pM – 100 uM
Target cell number		Number of target cells
T cell number		Number of PBMC x 0.6
CD3 per T cell		30,000 (Approximation base on literature and internaldata)
Target HLA per cell		332 for 5 nM pulse (Approximation based on internal epitope counting data)
Avogadro's number		6.0221409e+23
ImmTAC Diffusion coefficient	D	3.4e-8 (approximation that is too fast to allow meaningful depletion)
CD3 on rate	k_{on}^{CD3}	See Table S1
CD3 off rate	k_{off}^{CD3}	See Table S1
pHLA on rate	k_{on}^{HLA}	See Table S2
pHLA off rate	k_{off}^{HLA}	See Table S2
Time course	t	48 hrs (ELISA)<br>24 hrs (ELISpot)
Membrane binding rate modifier	σ	fitted
Kinetic proof-reading rate	k_p	fitted
Rate of dark state formation	k_i	fitted
Rate of dark state recovery	k_{rec}	fitted
Maximum IFNγ production	IFNmax	fitted
Sensitivity of IFNγ production	IFNC50	fitted

Supplementary Table 4 – 'Best particle' fitted parameters from different NY-BR-1 experiments rounded to 3 significant figures. LB (Lower bounds) and UB (Upper bounds) of fitting are shown on the left.

		Units	LB	UB	Figure 1	Figure 2
Max iteration:					25	20
Membrane binding rate modifier	σ	none	10^{-2}	10^{4}	0.294	0.074
Kinetic proof-reading rate	k_p	$\mu M^{-1} s^{-1}$	10^{-5}	10^{2}	0.0098	0.0023
Rate of dark state formation	k_i	$\mu M^{-1} s^{-1}$	10^{-4}	10^{4}	0.0102	0.097
Rate of dark state recovery	k_{rec}	$\mu M^{-1} s^{-1}$	10^{-15}	10^{-1}	0.00457	0.0000498
Maximum IFN γ production	IFNmax	$pg/ml\ s^{-1}$	10^{-2}	10^{4}	39	124
Sensitivity of IFNγ produ ction	IFNC50	μM	10^{-6}	10^{1}	1.23	0.97

Supplementary Table 5 - 'Best particle' fitted parameters from PRAME experiments. For HLA2+ cells mimetic copies expected on both T cells and cancer cells and starting values for local concentration of off-target mimetic fitted as if displayed uniformly on both Target cells and T cells. LB (Lower bounds) and UB (Upper bounds) of fitting are shown on the left.

		Units	LB	UB	Donor 1<br>Figure 6	Donor 2<br>Figure S4B	Donor 3<br>Figure S4C
					HLA-A201 +	HLA-A201 +	HLA-A201 -
Max iteration:					25	20	23
Membrane binding rate modifier	σ	none	10^{-2}	10^{2}	4.87	0.439	0.0159
Kinetic proof-reading rate	kp	$\mu M^{-1}\ s^{-1}$	10^{-5}	10^{0}	0.0013	0.00648	0.00143
Rate of dark state formation	ki	$\mu M^{-1}\ s^{-1}$	10^{-5}	10^{0}	0.0144	0.00182	0.218
Rate of dark state recovery	krec	$\mu M^{-1}\ s^{-1}$	10^{-8}	10^{-3}	2.67E-08	1.55E-06	5.33E-05
Maximum IFNγ production	IFNmax	pg/ml s^{-1}	10^{-3}	10^{4}	0.0144	0.0247	14.9
Sensitivity of IFNγ production	IFNC50	μM	10^{-7}	10^{1}	0.00000748	0.00129	0.00692
Mimetic off-rate		s^{-1}	10^{-4}	10^{0}	0.015	0.0108	0.319
Mimetic copies per cell		copies	10^{1}	10^{7}	86.3	155	375
Mimetic Affinity (μM) (kon fixed at 0.5 $\mu M^{-1}\ s^{-1}$)		μM			0.03	0.0216	0.638

Supplementary equations 1 – 10 ODE system:

[A] - Local ImmTAC in solution

$$\frac{d[A]}{dt} = -k_{on}^{CD3}[A][B] + k_{off}^{CD3}[C] - k_{on}^{HLA}[A][E] + k_{off}^{HLA}[D] - k_{off}^{CD3}[I] - k_{on}^{CD3}[A][J] - \frac{(D([A]-[Bulk]) \times area)}{activeVolume}$$

[B] - Free CD3

$$\frac{d[B]}{dt} = -k_{on}^{CD3}[A][B] + k_{off}^{CD3}[C] - \frac{k_{on}^{CD3}}{\sigma}[B][D] + k_{off}^{CD3}[F] + k_{off}^{CD3}[G] + k_{rec}[J]$$

[C] - CD3 bound to ImmTAC

$$\frac{d[C]}{dt} = k_{on}^{CD3}[A][B] - k_{off}^{CD3}[C] - \frac{k_{on}^{HLA}}{\sigma}[E][C] + k_{off}^{HLA}[F] + k_{off}^{HLA}[G] + k_{rec}[I]$$

[D] - Target HLA bound to ImmTAC

$$\frac{d[D]}{dt} = k_{on}^{HLA}[A][E] - k_{off}^{HLA}[D] - \frac{k_{on}^{CD3}}{\sigma}[B][D] + k_{off}^{CD3}[F] + k_{off}^{CD3}[G] + k_{off}^{CD3}[H] - \frac{k_{on}^{CD3}}{\sigma}[D][J]$$

[E] - Free target HLA

$$\frac{d[E]}{dt} = -k_{on}^{HLA}[A][E] + k_{off}^{HLA}[D] - \frac{k_{on}^{HLA}}{\sigma}[E][C] + k_{off}^{HLA}[F] + k_{off}^{HLA}[G] + k_{off}^{HLA}[H] - \frac{k_{on}^{HLA}}{\sigma}[E][I]$$

[F] - Fresh CD3-ImmTAC-target bridge

$$\frac{d[F]}{dt} = \frac{k_{on}^{HLA}}{\sigma}[E][C] - k_{off}^{HLA}[F] + \frac{k_{on}^{CD3}}{\sigma}[B][D] - k_{off}^{CD3}[F] - k_p[F]$$

[G] - Phosphorylated Bridges

$$\frac{d[G]}{dt} = k_p[F] - k_{off}^{HLA}[G] - k_{off}^{CD3}[G] - k_i[G]$$

[H] - Dark state target HLA bridge

$$\frac{d[H]}{dt} = k_i[G] - k_{off}^{HLA}[H] - k_{off}^{CD3}[H] + \frac{k_{on}^{HLA}}{\sigma}[E][I] + \frac{k_{on}^{CD3}}{\sigma}[J][D]$$

[I] - Dark state CD3 + IMMTAC-only

$$\frac{d[I]}{dt} = k_{off}^{HLA}[H] - k_{rec}[I] - k_{off}^{CD3}[I] + k_{on}^{CD3}[A][J] - \frac{k_{on}^{HLA}}{\sigma}[E][I]$$

[J] - Dark state CD3 only

$$\frac{d[J]}{dt} = k_{off}^{CD3}[I] - k_{rec}[J] - k_{on}^{CD3}[A][J] + k_{off}^{CD3}[H] - \frac{k_{on}^{CD3}}{\sigma}[J][D]$$

Supplementary equations 2 – 15 ODE system to define a system containing a cross-reactive mimetic:

[A] - Local ImmTAC in solution

$$\frac{d[A]}{dt} = -k_{on}^{CD3}[A][B] + k_{off}^{CD3}[C] - k_{on}^{HLA}[A][E] + k_{off}^{HLA}[D] - k_{off}^{CD3}[I] - k_{on}^{CD3}[A][J] - \frac{(D([A]-[Bulk]) \times area)}{activeVolume} - k_{on}^{mim}[A][K]$$

[B] - Free CD3

$$\frac{d[B]}{dt} = -k_{on}^{CD3}[A][B] + k_{off}^{CD3}[C] - \frac{k_{on}^{CD3}}{\sigma}[B][D] + k_{off}^{CD3}[F] + k_{off}^{CD3}[G] + k_{rec}[J] - \frac{k_{on}^{CD3}}{\sigma}[B][L] + k_{off}^{CD3}[M] + k_{off}^{CD3}[N]$$

[C] - CD3 bound to ImmTAC

$$\frac{d[C]}{dt} = k_{on}^{CD3}[A][B] - k_{off}^{CD3}[C] - \frac{k_{on}^{HLA}}{\sigma}[E][C] + k_{off}^{HLA}[F] + k_{off}^{HLA}[G] + k_{rec}[I] - \frac{k_{on}^{mim}}{\sigma}[K][C] + k_{off}^{mim}[M] + k_{off}^{mim}[N]$$

[D] - Target HLA bound to ImmTAC

$$\frac{d[D]}{dt} = k_{on}^{HLA}[A][E] - k_{off}^{HLA}[D] - \frac{k_{on}^{CD3}}{\sigma}[B][D] + k_{off}^{CD3}[F] + k_{off}^{CD3}[G] + k_{off}^{CD3}[H] - \frac{k_{on}^{CD3}}{\sigma}[D][J]$$

[E] - Free target HLA

$$\frac{d[E]}{dt} = -k_{on}^{HLA}[A][E] + k_{off}^{HLA}[D] - \frac{k_{on}^{HLA}}{\sigma}[E][C] + k_{off}^{HLA}[F] + k_{off}^{HLA}[G] + k_{off}^{HLA}[H] - \frac{k_{on}^{HLA}}{\sigma}[E][I]$$

[F] - Fresh CD3-ImmTAC-target bridge

$$\frac{d[F]}{dt} = \frac{k_{on}^{HLA}}{\sigma}[E][C] - k_{off}^{HLA}[F] + \frac{k_{on}^{CD3}}{\sigma}[B][D] - k_{off}^{CD3}[F] - k_p[F]$$

[G] - Phosphorylated target Bridges

$$\frac{d[G]}{dt} = k_p[F] - k_{off}^{HLA}[G] - k_{off}^{CD3}[G] - k_i[G]$$

[H] - Dark state target HLA bridge

$$\frac{d[H]}{dt} = k_i[G] - k_{off}^{HLA}[H] - k_{off}^{CD3}[H] + \frac{k_{on}^{HLA}}{\sigma}[E][I] + \frac{k_{on}^{CD3}}{\sigma}[J][D]$$

[I] - Dark state CD3 + IMMTAC-only

$$\frac{d[I]}{dt} = k_{off}^{HLA}[H] - k_{rec}[I] - k_{off}^{CD3}[I] + k_{on}^{CD3}[A][J] - \frac{k_{on}^{HLA}}{\sigma}[E][I] + k_{off}^{mim}[O] - \frac{k_{on}^{mim}}{\sigma}[K][I]$$

[J] - Dark state CD3 only

$$\frac{d[J]}{dt} = k_{off}^{CD3}[I] - k_{rec}[J] - k_{on}^{CD3}[A][J] + k_{off}^{CD3}[H] - \frac{k_{on}^{CD3}}{\sigma}[J][D] - \frac{k_{on}^{CD3}}{\sigma}[J][L] + k_{off}^{CD3}[O]$$

[K] - Free mimetic HLA

$$\frac{d[K]}{dt} = -k_{on}^{mim}[A][K] + k_{off}^{mim}[L] - \frac{k_{on}^{mim}}{\sigma}[K][C] + k_{off}^{mim}[M] + k_{off}^{mim}[N] + k_{off}^{mim}[O] - \frac{k_{on}^{mim}}{\sigma}[K][I]$$

[L] - Mimetic HLA bound to IMMTAC

$$\frac{d[L]}{dt} = k_{on}^{mim}[A][K] - k_{off}^{mim}[L] - \frac{k_{on}^{CD3}}{\sigma}[B][L] + k_{off}^{CD3}[M] + k_{off}^{CD3}[N] + k_{off}^{CD3}[O] - \frac{k_{on}^{CD3}}{\sigma}[L][J]$$

[M] - Mimetic HLA bound to IMMTAC bound to receptor

$$\frac{d[M]}{dt} = \frac{k_{on}^{mim}}{\sigma}[K][C] - k_{off}^{mim}[M] + \frac{k_{on}^{CD3}}{\sigma}[B][L] - k_{off}^{CD3}[M] - k_p[M]$$

[N] - Signalling mimetic bridge

$$\frac{d[N]}{dt} = k_p[M] - k_{off}^{mim}[N] - k_{off}^{CD3}[N] - k_i[N]$$

[O] - Dark state Bridge to mimetic HLA

$$\frac{d[O]}{dt} = k_i[N] - k_{off}^{mim}[O] - k_{off}^{CD3}[O] + \frac{k_{on}^{mim}}{\sigma}[K][I] + \frac{k_{on}^{CD3}}{\sigma}[J][L]$$

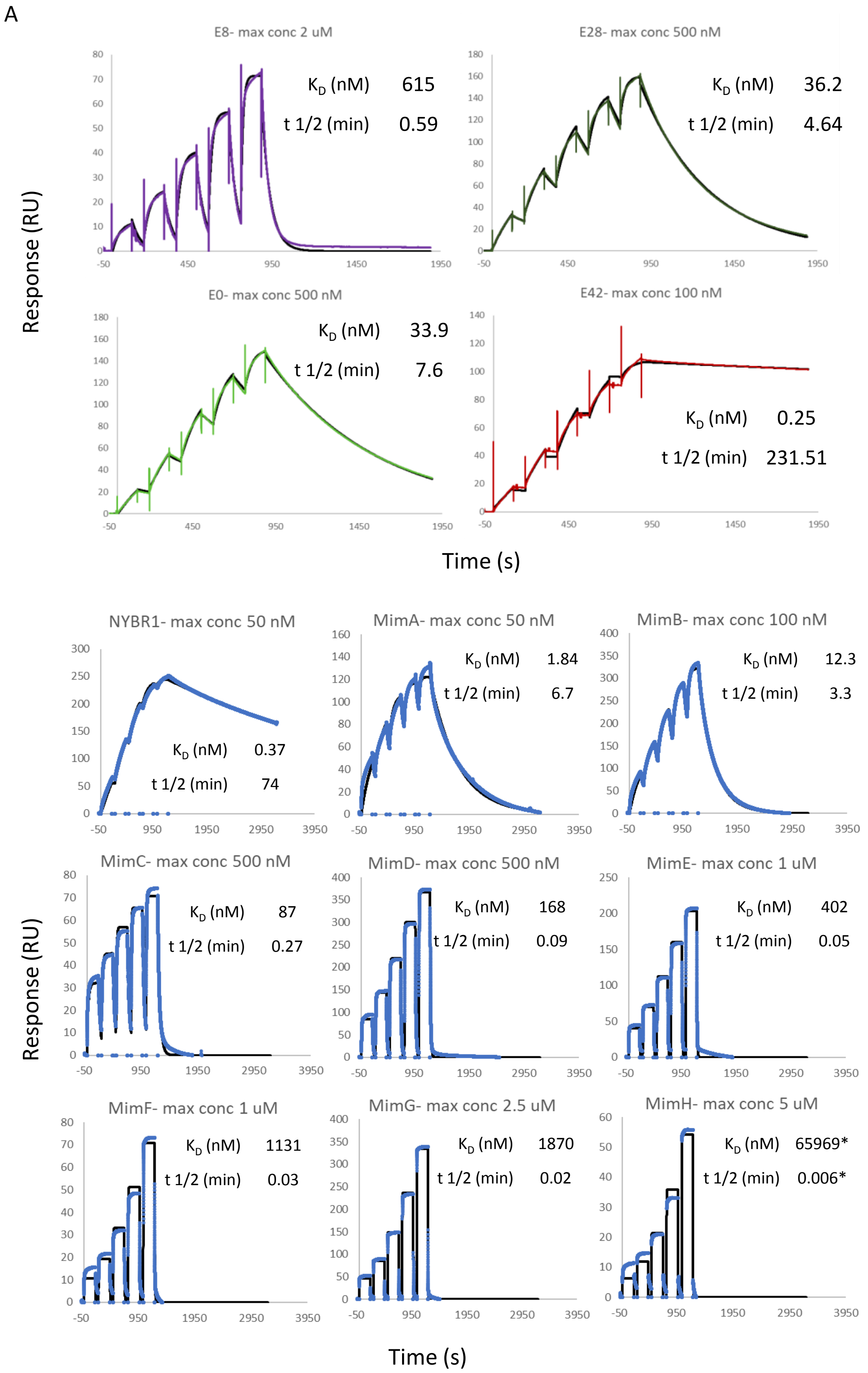

Supplementary Figure S1 – Key SPR data. A) Single cycle Biacore data for key anti-CD3 variants in the context of anti-NYBR1 ImmTAC molecules binding to human CD3εδ (ACRO biosystems) amine coupled to CM5 sensor chip at 37 °C in PBS pH 7.4 with 0.005% P20. Five 2x serial dilutions were injected with the highest concentration listed above each graph. Data is coloured according to variants and kinetic fits are shown with a black line. Data was collected with a Biacore T200 instrument. **B)** Single cycle Biacore data for anti-NYBR1 TCR binding to different biotinylated pHLA at 37 °C in PBS pH 7.4 with 0.005% P20. pHLA were immobilised via streptavidin that was amine coupled to a CM5 sensor chip and analyses carried out using a Biacore 8K instrument. *KD value determined by equilibrium fit and t1/2 based on separate fitting of off-rate, likely inaccurate due to the rapid dissociation observed.

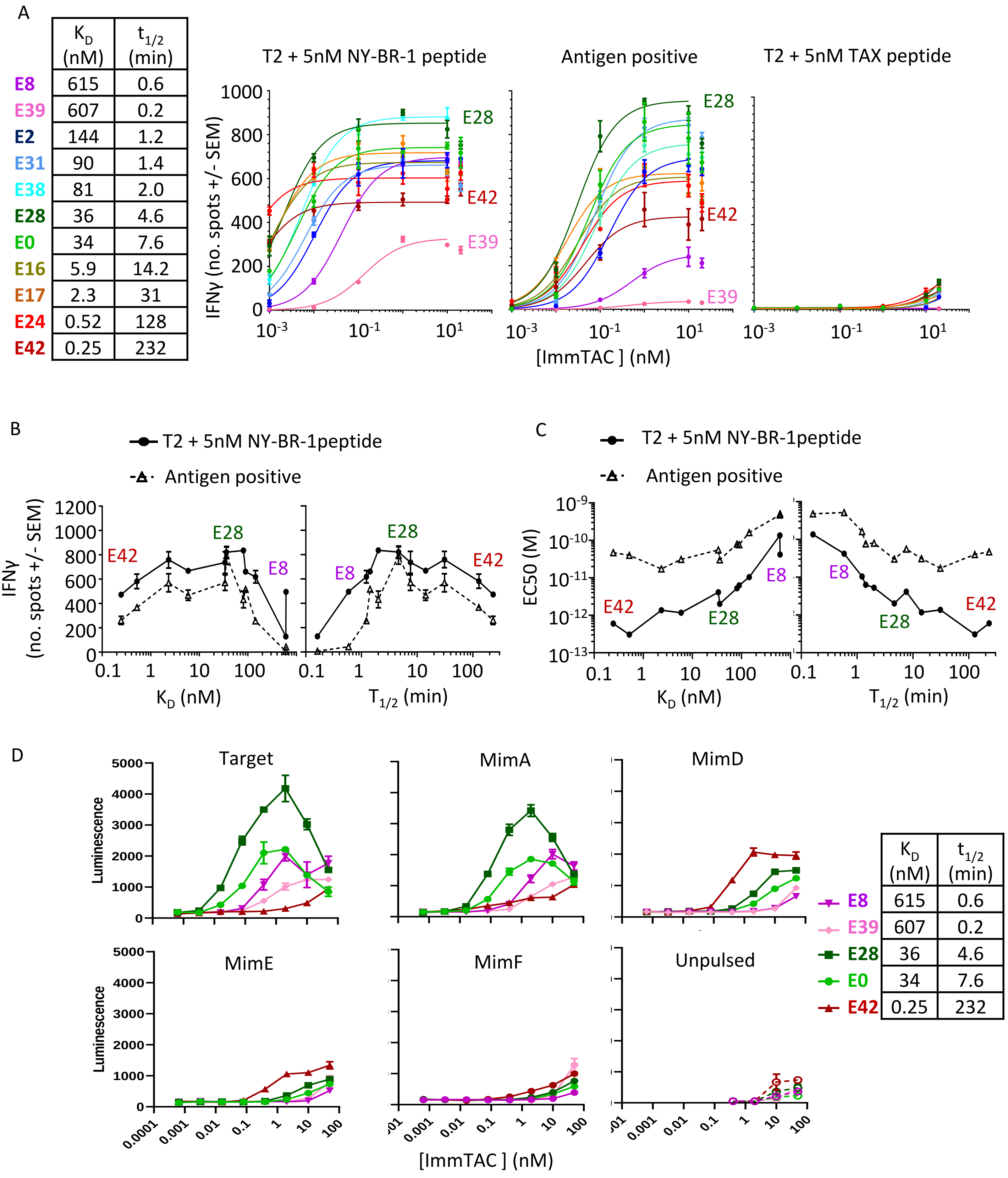

	K_D (nM)	$t_{1/2}$ (min)
E8	615	0.6
E39	607	0.2
E2	144	1.2
E31	90	1.4
E38	81	2.0
E28	36	4.6
E0	34	7.6
E16	5.9	14.2
E17	2.3	31
E24	0.52	128
E42	0.25	232

	K_D (nM)	$t_{1/2}$ (min)
E8	615	0.6
E39	607	0.2
E28	36	4.6
E0	34	7.6
E42	0.25	232

Supplementary Figure S2 – Effect of CD3 affinity on ImmTAC activity with a highly specific TCR (NY-BR-1)
A) IFNγ production measured by ELISpot from HLA-A*02:01 negative PBMCs in the presence of ImmTAC molecules with different anti-CD3 affinities from, left: T2 cells pulsed with 5 nM NY-BR-1 peptide, centre: antigen positive CAMA-1 A2B2M cells, or right: T2 cells pulsed with irrelevant TAX peptide. All data points representative of 3 experimental repeats. **B)** Summary plots of ELISpot data showing spots at 0.1 nM ImmTAC plotted against CD3 binding affinity or $t_{1/2}$. **C)** Summary plots of EC_{50} plotted against CD3 binding affinity or $t_{1/2}$. **D)** Activation assays with jurkat NFAT-luciferase reporter cell line (Promega) incubated for 22 hours with T2 cells pulsed with 1 nM target or mimetic peptides.

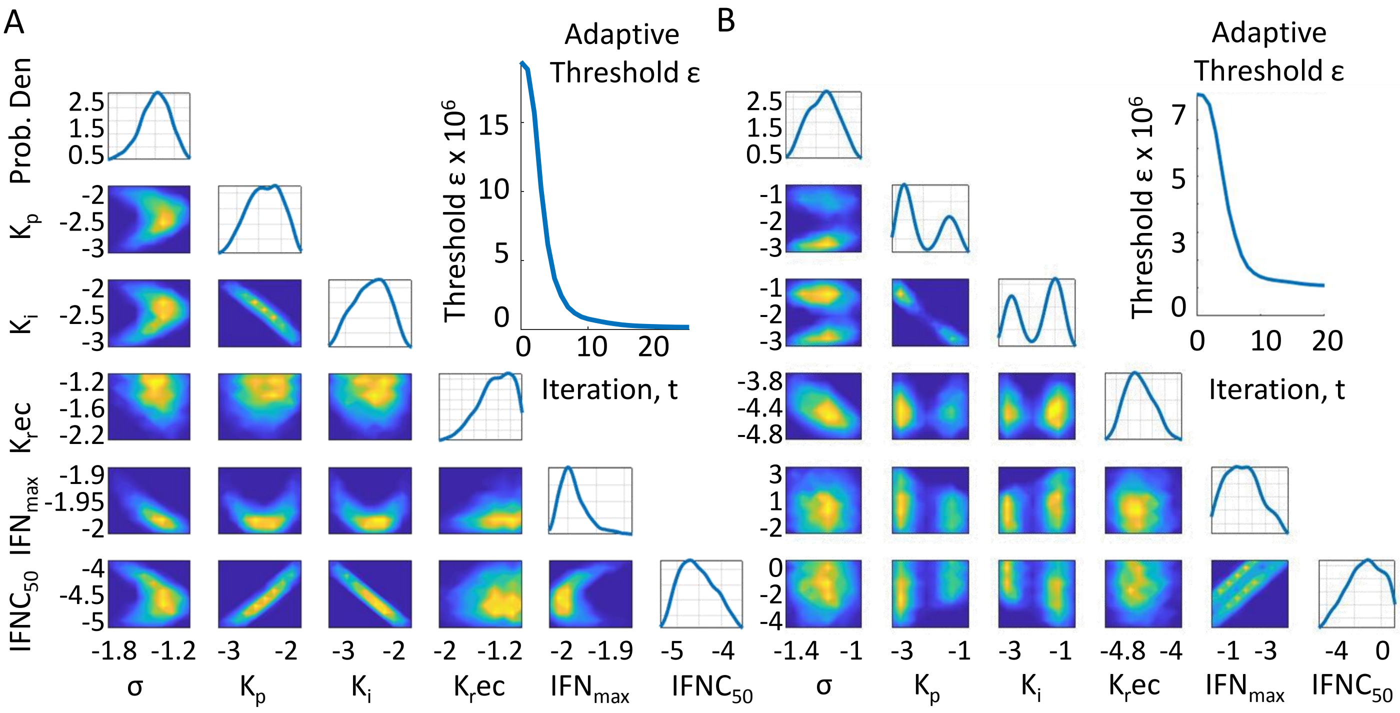

Supplementary Figure S3 – Probability distributions for Model fitting. A) Probability distributions for model parameters at final iteration of ABC-SMC fit shown in figure 4A, with values expressed as log values, ie 10 ^ (value on axis) and best particles values listed in table S1. **B)** Parameters distributions for fit to data shown in Figure 4B. A single optimum parameter set was not obtained here but two general solutions seem possible to fit the data. One with a faster kinetic proof-reading rate and a slower rate of dark state formation and the other with a fast dark-state formation rate and slower kinetic proof-reading rate. The fits to the data obtained with either parameter set both appear visually very similar and the same outlier data points are observed (Not shown).

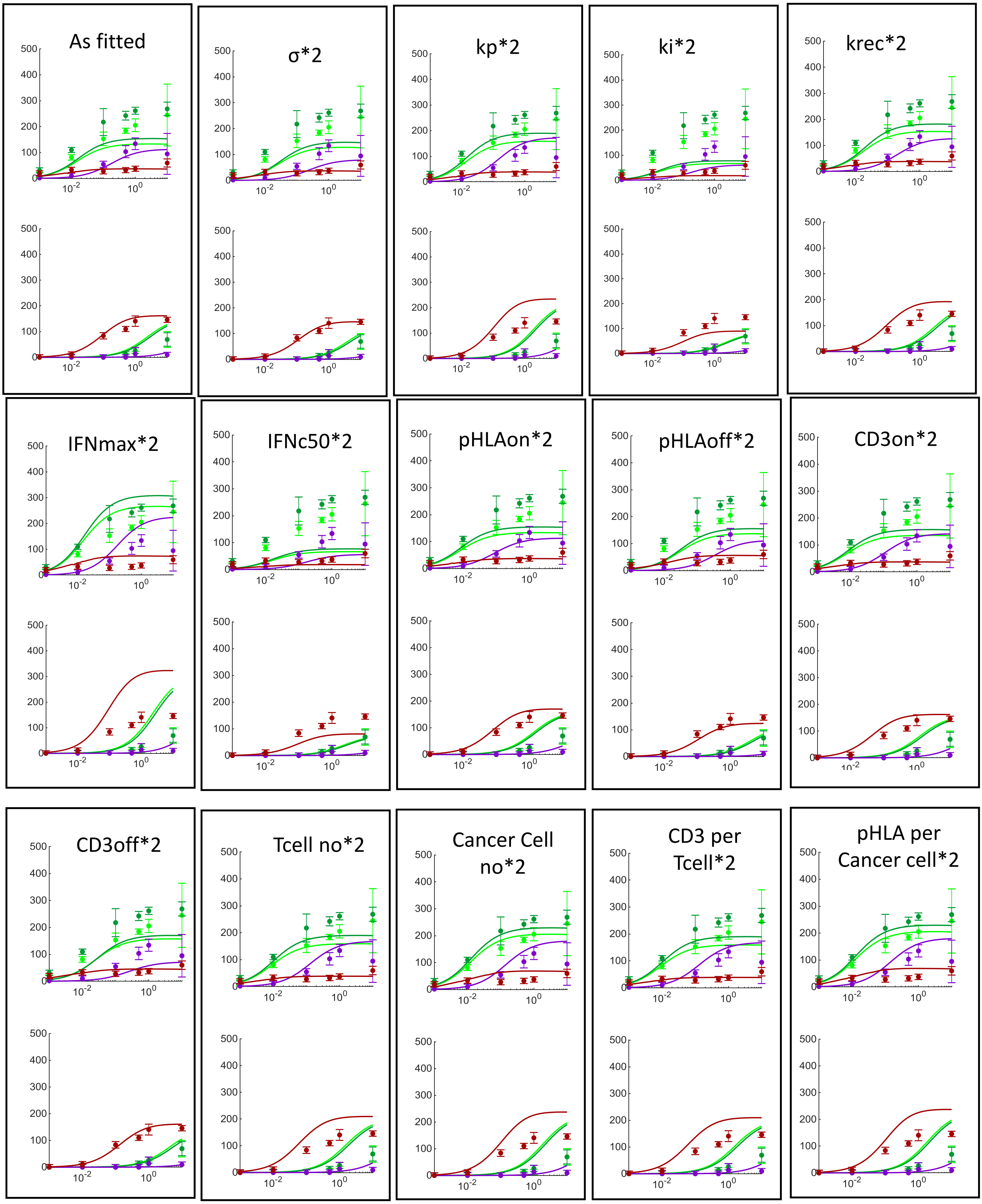

Supplementary Figure S4– Effect of 2x change in fitted and fixed parameters on model output for NYBR1 Target and MimC. To assess the sensitivity of the model to fitted and non-fitted parameters simulations were performed doubling the value of one parameter at a time and results are shown for target (top) and MimC (bottom). Changing some parameters appears to improve the fit with NYBR1-target reactivity but gives a worse fit with MimC. Generally however parameter changes on this scale do not significantly alter the output of the model and the trends remain the same.

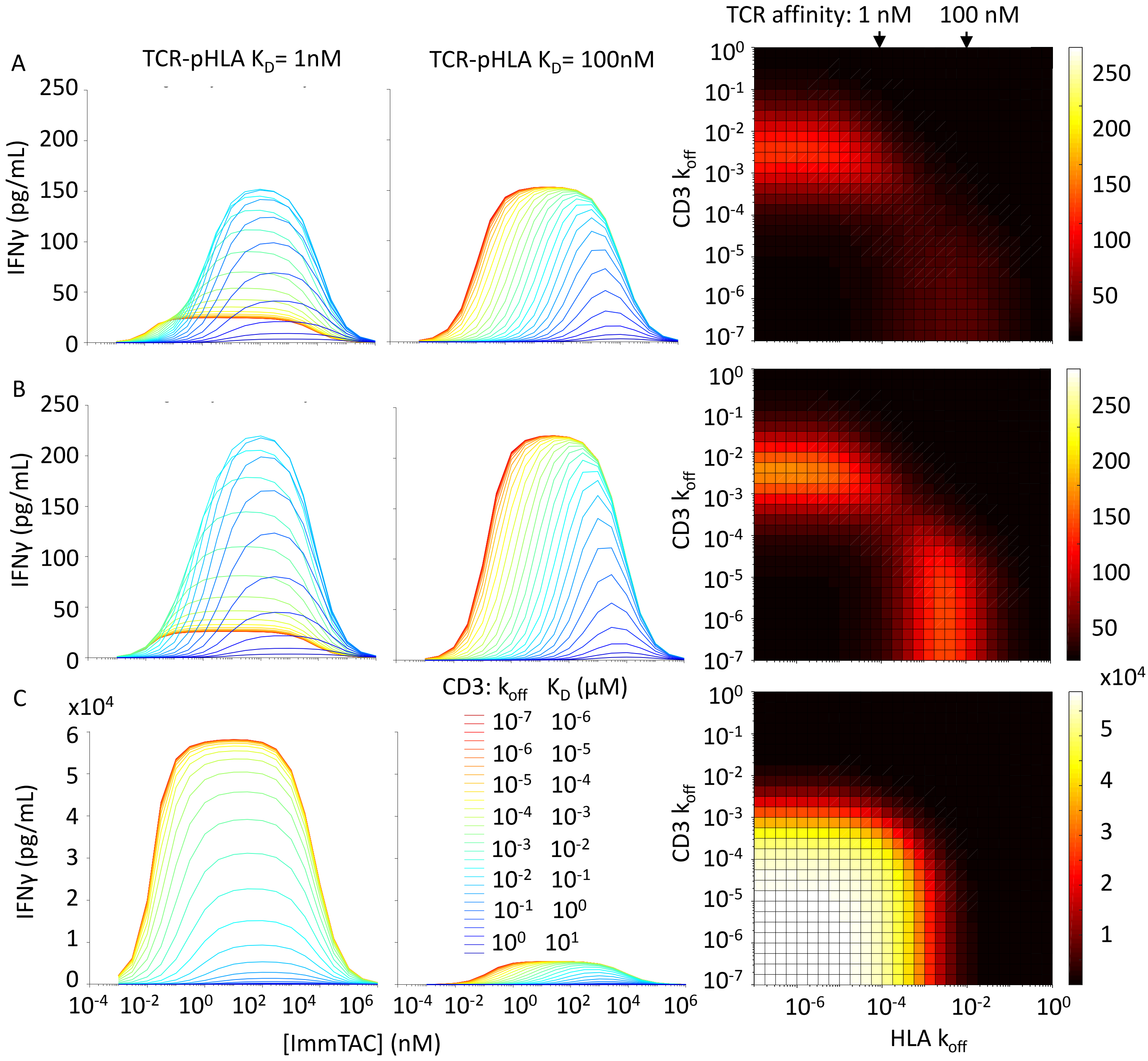

Supplementary Figure S5– Effect of altering key model parameters. A) Result of simulation using parameter fits from Figure 4B (Table S4) showing the effects of varying CD3 off-rate. Left: strong TCR-pHLA binding (K_D= 1nM), middle: intermediate TCR-pHLA binding (K_D= 100nM), and right: a heat map of response at 10 pM ImmTAC with different TCR-pHLA or CD3 affinity combinations. **B)** Effect of using same parameters as in A, but making dark-state recovery extremely fast when released from a complex (k_{rec} = 10^{15}). While overall response only increases slightly the main difference is that the response with intermediate TCR-pHLA : strong CD3 binding at 10 pM is more similar to that seen with strong TCR-pHLA : intermediate CD3 binding. **C)** Effect of using same parameters as in A, but making dark-state formation extremely slow (k_i= 10^{-30}). Without dark-state formation a considerably higher response is generated with both strong and intermediate affinity TCR-pHLA binding, and strong TCR-pHLA : strong CD3 binding generates the highest responses. CD3 off-rates in all figures here range from 10^{-7} s^{-1} (red) to 10^{0} (blue) with a constant on-rate of 0.1 μM^{-1} s^{-1}.

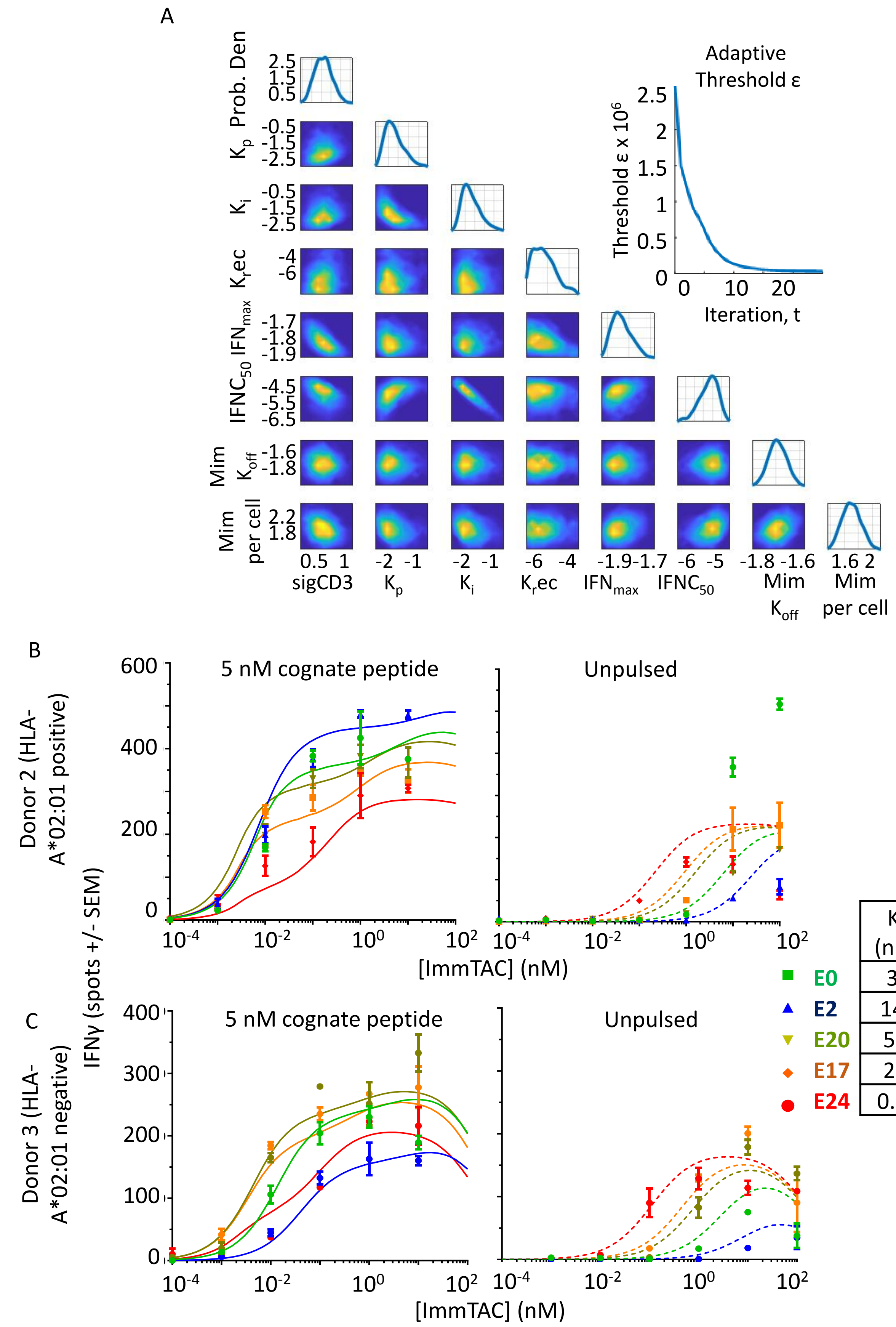

	K_D (nM)	$t_{1/2}$ (min)
E0	34	7.6
E2	144	1.2
E20	5.5	10
E17	2.3	31
E24	0.52	128

Supplementary Figure S6– ABC-SMC fitting data for cross-reactivity model applied to tool ImmTAC with 'TCR-X'. A) Parameter distributions obtained when fitting ELISpot data in Figure 6 B. Parameters expressed as log values, (ie 10^ value on axis) and best particle values are listed in Table S5. **B)** ELISpot of IFNγ production from a different PBMC donor (donor 2). Lines show model fits with parameters obtained in Table S5. **C)** ELISpot of IFNγ production from a third, HLA A*02:01 negative, PBMC donor (donor 3). Lines show model fits with parameters obtained in Table S5.

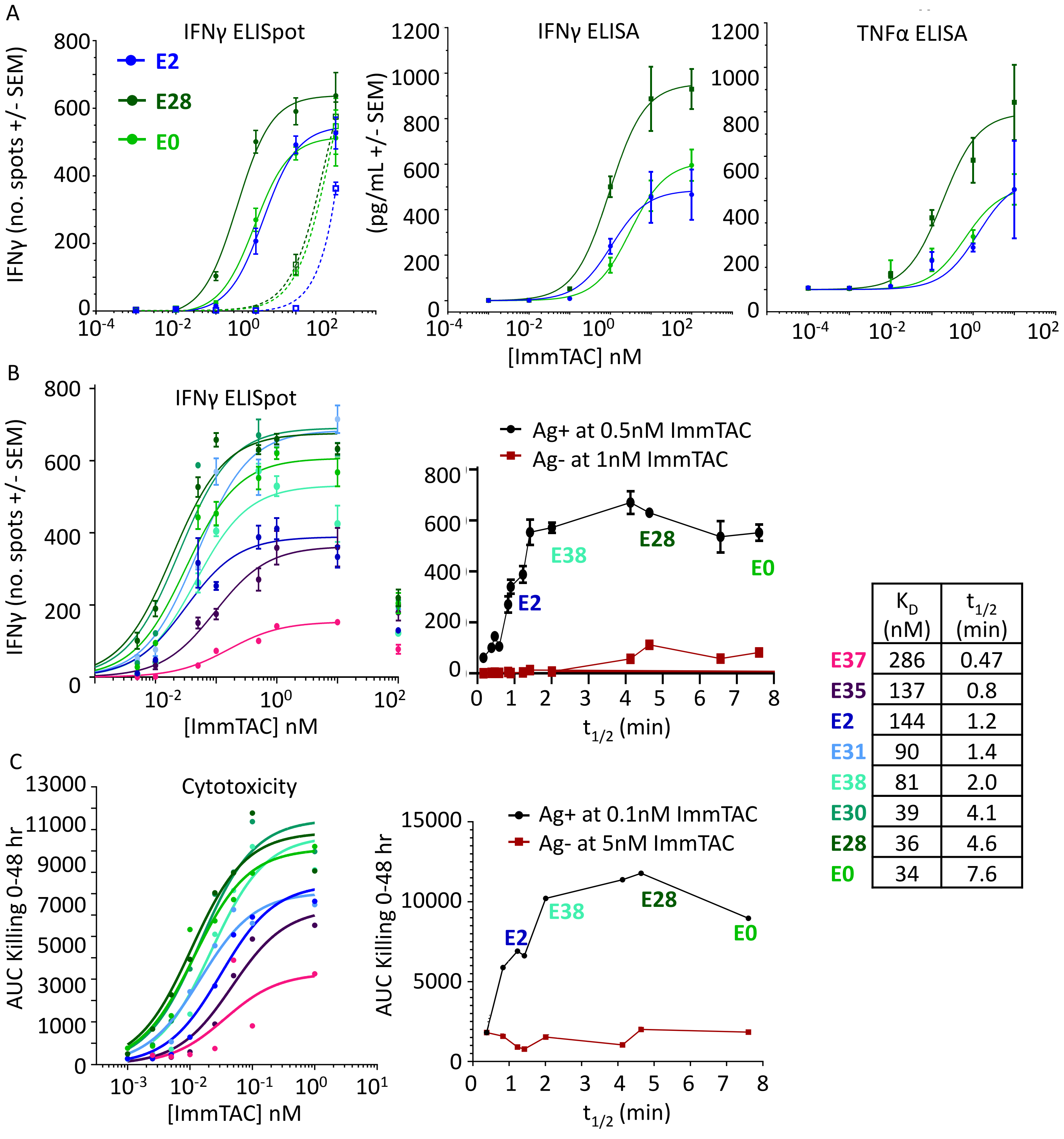

	K_D (nM)	$t_{1/2}$ (min)
E37	286	0.47
E35	137	0.8
E2	144	1.2
E31	90	1.4
E38	81	2.0
E30	39	4.1
E28	36	4.6
E0	34	7.6

Supplementary Figure S7 – Confirming optimal anti-CD3 kinetics on tool 'TCR-X' in cytokine release and cytotoxicity assays. A) Focused comparison of E0, E28 and E2 variants. PBMCs (HLA-A*02:01 positive) incubated with antigen positive Mel624 cells (filled circles with solid lines) or antigen negative Granta519 cells (open squares with dashed lines). Curves represent 3-parameter fits to data obtained by IFNγ ELISpot (left), IFNγ MSD ELISA (middle), and TNFα MSD ELISA. **B)** Testing a focused panel of nanomolar affinity anti-CD3 variants: ELISpot on Antigen positive MEL624 cell line, with each data point performed in triplicate. The E28 variant was included on every plate and all data was normalised to the result obtained with this variant. Curves represent simple 3-parameter fits of data up to 10 nM ImmTAC. On the right the responses at specific ImmTAC concentrations are plotted against $T_{1/2}$ and also includes response to antigen negative MDAMB231 cell line. **C)** Incucyte killing assays of the same focused panel with areas under the curves up to 48 hours plotted against ImmTAC concentration. On the right AUC at a specified ImmTAC concentration is plotted against $T_{1/2}$ of CD3 binding, including response to antigen negative MDAMB231 cell line.

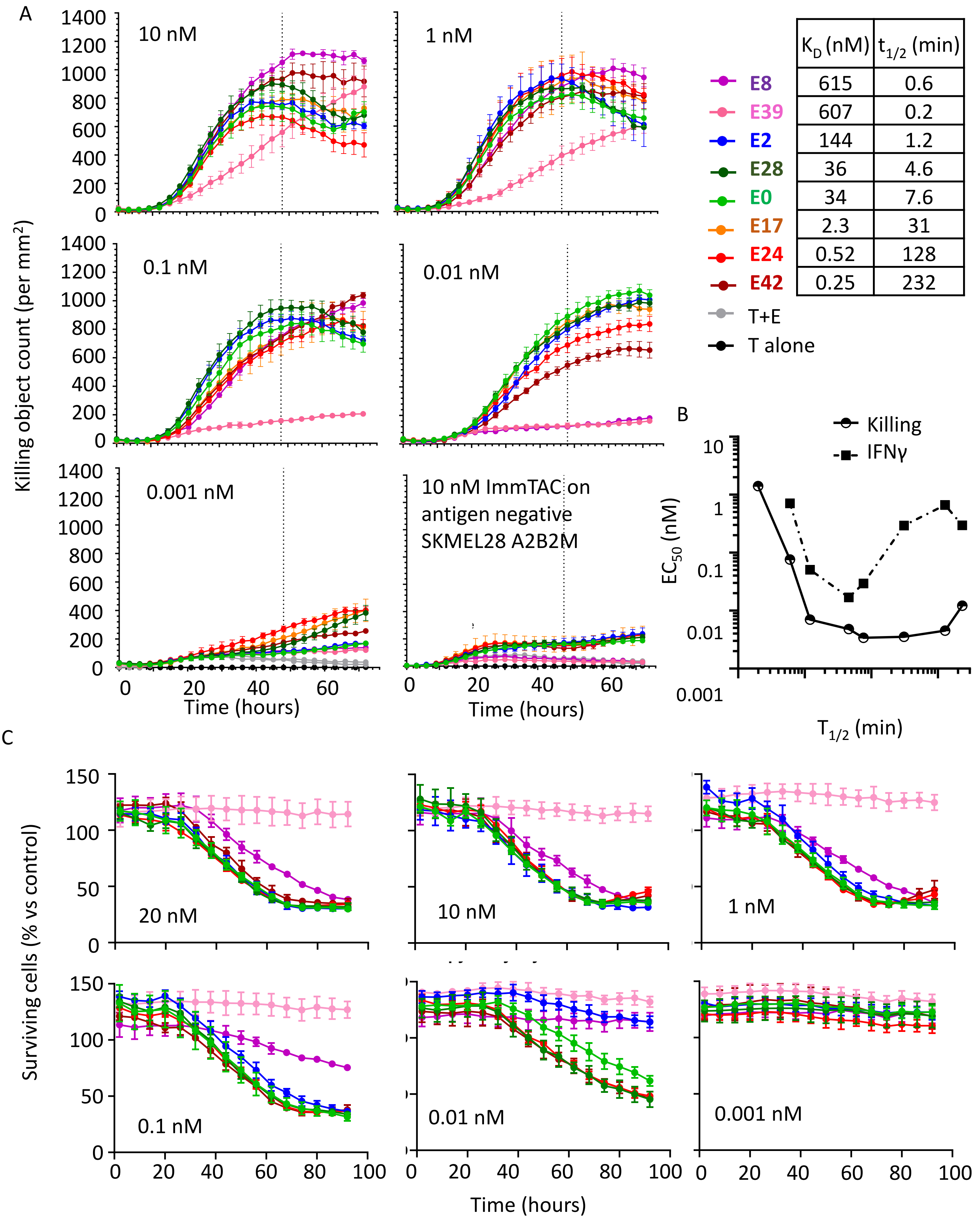

	K_D (nM)	$t_{1/2}$ (min)
E8	615	0.6
E39	607	0.2
E2	144	1.2
E28	36	4.6
E0	34	7.6
E17	2.3	31
E24	0.52	128
E42	0.25	232

Supplementary Figure S8 – Effect of CD3 affinity on killing of NYBR1 positive cells. A) Killing time courses as measured by Incucyte assay with 8 different anti-CD3 variants. 5 panels show results on CAMA-1 A2B2M antigen positive cells with ImmTAC concentration shown top left, and one panel (bottom right) shows killing with the SKMEL28 A2B2M antigen negative cell line at 10 nM ImmTAC. **B)** Plot of EC_{50} for IFNγ release against $t_{1/2}$ of CD3 binding. **C)** Data from a separate killing experiment using the Phenix instrument where here the % of surviving target cells is plotted relative to control target growth without ImmTAC.
